# α-Arrestins and Their Functions: From Yeast to Human Health

**DOI:** 10.3390/ijms23094988

**Published:** 2022-04-30

**Authors:** Kacper Zbieralski, Donata Wawrzycka

**Affiliations:** Department of Genetics and Cell Physiology, University of Wrocław, Kanonia 6/8, 50-328 Wrocław, Poland; kacper.zbieralski2@uwr.edu.pl

**Keywords:** α-arrestin, ARTs, yeast, endocytosis, protein trafficking adaptors, membrane transporters

## Abstract

α-Arrestins, also called arrestin-related trafficking adaptors (ARTs), constitute a large family of proteins conserved from yeast to humans. Despite their evolutionary precedence over their extensively studied relatives of the β-arrestin family, α-arrestins have been discovered relatively recently, and thus their properties are mostly unexplored. The predominant function of α-arrestins is the selective identification of membrane proteins for ubiquitination and degradation, which is an important element in maintaining membrane protein homeostasis as well as global cellular metabolisms. Among members of the arrestin clan, only α-arrestins possess PY motifs that allow canonical binding to WW domains of Rsp5/NEDD4 ubiquitin ligases and the subsequent ubiquitination of membrane proteins leading to their vacuolar/lysosomal degradation. The molecular mechanisms of the selective substrate’s targeting, function, and regulation of α-arrestins in response to different stimuli remain incompletely understood. Several functions of α-arrestins in animal models have been recently characterized, including redox homeostasis regulation, innate immune response regulation, and tumor suppression. However, the molecular mechanisms of α-arrestin regulation and substrate interactions are mainly based on observations from the yeast *Saccharomyces cerevisiae* model. Nonetheless, α-arrestins have been implicated in health disorders such as diabetes, cardiovascular diseases, neurodegenerative disorders, and tumor progression, placing them in the group of potential therapeutic targets.

## 1. Introduction

In the late 1980s, studies on a G-protein coupled receptor (GPCR) rhodopsin contributed to the characterization of a 48 kDa protein arrestin (arrestin-1), named after its ability to inhibit rhodopsin’s signal transduction (“signal arrest”) [1]. Subsequent studies have identified and characterized the remaining members of the visual/β-arrestin family proteins in humans as holding a total number of four isoforms: visual arrestins (arrestin-1/Arr1, arrestin-4/Arr4), named in reference to rhodopsin, and β-arrestins (arrestin-2/Arr2/β-arrestin-1, arrestin-3/Arr3/β-arrestin-2), named regarding the β2-adrenergic receptor (β2-AR) [2]. The family of visual/ β-arrestins has soon turned out to participate in a plethora of cellular processes in animal cells, for instance, as GPCR activity regulators (reviewed in [3]), scaffold proteins involved in cellular signaling (reviewed in [4]) with a vast range of potential interactions partners [5], endocytic adaptors for clathrin-mediated endocytosis [6], and adaptor proteins for the NEDD4-like ubiquitin ligases involved in the ubiquitination of various plasma membrane (PM) proteins [7,8,9,10]. In the interim, arrestin-related trafficking (ARTs) adaptors, an emerging group of proteins that resemble β-arrestins, have been discovered [11]. Multiple studies, especially in fungi, have demonstrated their ability to interact with NEDD4-like ligases such as the *Saccharomyces cerevisiae* Rsp5 ligase that allows ubiquitination of various PM proteins and their subsequent vacuole/lysosome trafficking [11,12,13,14]. A phylogenetic analysis of visual arrestins, β-arrestins, and newly identified ARTs demonstrated that ARTs are indeed ubiquitous proteins conserved from yeast to humans, and that both families, together with the Vps26 family in eukaryotes and the Spo0M family in bacteria and archaea, constitute the arrestin clan [2]. Interestingly, β-arrestins seem to have diverged from α-arrestins relatively recently, which in fact makes them the youngest members of the arrestin clan [2]. Given ARTs’ evolutionary primacy, and to distinguish them from closely related visual/β-arrestins as well as other arrestin-like proteins, it has been proposed to redesignate ARTs as α-arrestins [2]. Most of the data on the molecular mechanisms and function of α-arrestins come from studies of the budding yeast model. Despite a limited knowledge of their biology, α-arrestins have been already linked to a range of cellular processes and several major health disorders, such as diabetes [15,16], cardiovascular diseases [17], neurological disorders [18], and tumor progression [19,20,21], which places them in the group of potential therapeutic targets.

## 2. Conserved Aspects of α-Arrestin Biology

**Figure 1:**
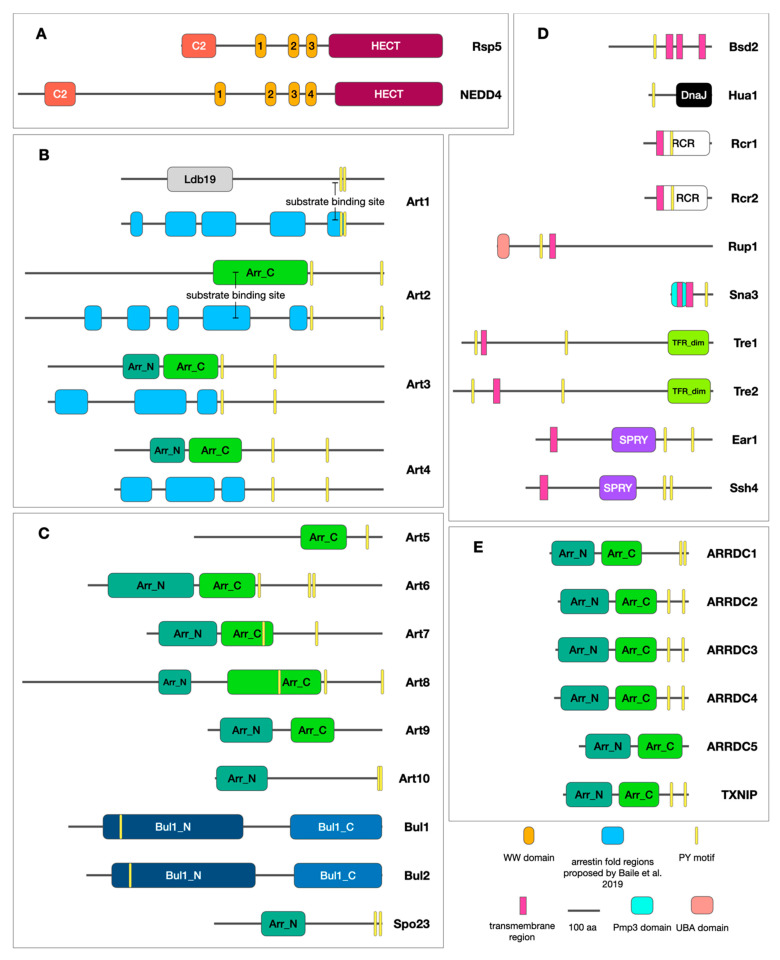
Schematic representation of the domain structure for the proteins of the Rsp5/NEDD4–adaptor complexes. (**A**) Schematic representation of the domain structure for the yeast Rsp5 and human NEDD4 ubiquitin ligases. (**B**) Schematic representation and comparison of predicted arrestin-N (Arr_N), arrestin-N-like (Ldb19), and arrestin-C (Arr_C) domains as defined in the Pfam database [25] and arrestin fold regions as proposed by Baile et al. 2019 (arrestin domain disrupted by multiple insertions) [26]; for yeast α-arrestins Art1-4; known substrate binding sites were indicated. (**C**) Schematic representation of domain structure for yeast α-arrestins Art5-10, Bul1-2, and Spo23. (**D**) Schematic representation of domain structure for yeast non-arrestin adaptors of the Rsp5 ligase. (**E**) Schematic representation of domain structure for human α-arrestins. Accession numbers for predicted domains as defined in Pfam database: Ldb19 (PF13002), Arr_N (PF00339), Arr_C (PF02752), Bul1_N (PF04425), Bul1_C (PF04426), DnaJ (PF00226), UBA (PF00627), Pmp3 (PF01679), TFR_dim (PF04253), SPRY (PF00622).

The arrestin clan members share a characteristic arrestin fold, divided into two arrestin domains of a β-sandwich structure connected by a hinge domain [11,22,23,24]. The main differences that separate α-arrestins from β-arrestins are the absence of the N-terminal helix as well as binding sites for clathrin, AP-2 complexes, and phosphoinsitides, which are typical for β-arrestins [2]. Moreover, α-arrestins are the only arrestin clan members that are known to possess [L/P]PxY (PY) motifs, which allow canonical binding to the Rsp5/NEDD4 ligases (Figure 1A–C,E) [2].

In humans, only six α-arrestins have been identified: five arrestin domain-containing proteins (ARRDC1-5) and a thioredoxin-interacting protein (TXNIP) (Figure 1E) [2]. Human α-arrestins display both structural and functional similarities to their yeast homologs. Intriguingly, recent studies on their tertiary structure have provided evidence for the presence of interspersed disordered loops in arrestin fold variants of the yeast Art1 α-arrestin (see Figure 1B for alternative domain structures of yeast Art1-4) [26], although it remains unclear whether human α-arrestins possess similar structural properties. Nonetheless, all human α-arrestins but one (ARRDC5) possess a pair of C-terminal PY motifs [2], which indicate interactions with the NEDD4-like family ligases. NEDD4, the presumably ancestral member of the family in humans, shows the greatest homology to the yeast Rsp5 ubiquitin ligase [27]. The Rsp5/NEDD4 family ligases recognize their substrates through WW domains (Figure 1A), which bind polyproline PY motifs [28,29] or a phosphorylated serine/threonine adjacent to a proline residue [30] in substrates. Given these circumstances, α-arrestins are ideal candidates for adaptor proteins involved in proteome regulation via NEDD4-mediated ubiquitination. Indeed, yeast α-arrestins cooperate with Rsp5 ubiquitin ligase [11], and human α-arrestins have been reported to interact with several members of the NEDD4 family [7,31,32,33,34].

Similarly to β-arrestins, members of the α-arrestin family in humans are widely expressed across human tissues [35]. Their expression pattern has been also reported to vary in major health disorders such as asthma [36], chronic obstructive pulmonary disease (COPD) [37] or several types of cancer [19,34,38], indicating their important role in the proper functioning of an organism. As their yeast homologs (Table 1), human α-arrestins have been shown to localize in distinct subcellular compartments. Most of the human α-arrestins localize to the PM and cytoplasm [7,31,39], although they are also found in cytoplasmic vesicles, endosomes, and lysosomes, as they participate in cargo endocytosis [7,39,40], as well as in ectosomes in the case of ARRDC1 protein [41]. Uniquely, TXNIP has been found to also localize in the nucleus [42,43] from where, in response to oxidative stress, it is translocated to mitochondria [42].

## 3. Nature of α-Arrestins—A Lesson from Yeast

### 3.1. Function of Yeast α-Arrestins

Yeast is a convenient model organism for the study of human proteins because many proteins are conserved from yeast to humans [87]. α-Arrestins have been particularly studied in the yeast *S. cerevisiae*, although many aspects of their biology remain elusive. In budding yeast, 14 α-arrestins have been recognized, including 10 proteins of the Art family (Art1/Ldb19 [11], Art2/Ecm21 [11], Art3/Aly2 [11], Art4/Rod1 [11], Art5 [11], Art6/Aly1 [11], Art7/Rog3 [11], Art8/Crs2 [11], Art9/Rim8 [11] and Art10 [13]), three distantly related Bul (binds ubiquitin ligase) family members (Bul1-3) [14] and the Spo23 protein [24] (Table 1). A single *VPS26* gene and no β-arrestins were identified in yeast [2]. 

Yeast α-arrestins have been primarily known as adaptor proteins for the Rsp5 ligase-mediated ubiquitination of PM proteins. Rsp5 is the sole NEDD4 family member in *S. cerevisiae* and the only E3 enzyme known to target PM proteins in yeast [28]. The Rsp5/NEDD4 family representatives usually hold two to four WW domains, which serve as substrate binding sites [27] (Figure 1A), although only a few PM proteins possess motifs recognized by the Rsp5 ligase. Instead, Rsp5 cooperates with α-arrestins, which can recognize specific degradation signals in PM substrates and recruit Rsp5 for their ubiquitination in response to various physiological (ligand- or substrate-binding [49,88]) and stress (heat stress [89]), starvation [90], and antibiotic exposure [51]) stimuli. In 1996, studies on glutathione *S*-transferase-dependent drug resistance in yeast led to the identification of Rod1 and Rog3 proteins, which when overexpressed accounted for *o*-dinitrobenzene (*o*-DNB), zinc, and calcium resistance in *S. cerevisiae* [60]. The possible mechanism of *o*-DNB tolerance provided by these proteins was suggested to depend on ABC (ATP-binding cassette) transporter regulation in cooperation with the Rsp5 ligase [91]. In 2008, Lin et al. performed an analysis, which revealed other putative yeast α-arrestins able to interact with Rsp5 [11]. For instance, the role of Art1 as an adaptor for the Rsp5-dependent ubiquitination of the arginine permease Can1’s degradation has been established [11]. Furthermore, Art1, together with Art2, have been shown to target the lysine permease Lyp1 as well, although their involvement in Lyp1 ubiquitination depended on different degradation stimuli [11]. The studies have provided significant evidence for yeast α-arrestins to facilitate Rsp5 cargo recognition, and their involvement in this process has been comprehensively investigated ever since then. Consequently, yeast α-arrestins have been found to facilitate Rsp5-dependent ubiquitination and endocytosis of various PM transporters, including the general amino acid permease Gap1 [53,80,88], monocarboxylate transporter Jen1 [64], arginine permease Can1 [11,45], methionine transporter Mup1 [11], divalent metal ion transporter Smf1 [50], inositol transporter Itr1 [13], and uracil transporter Fur4 [50] (Table 1). Selective endocytosis followed by lysosomal degradation is a main mechanism of PM transporter downregulation in response to specific environmental signals [45,51,62]. The quantitative proteomics study demonstrated that under cycloheximide-induced stress conditions, many previously unexamined PM proteins undergo α-arrestin-dependent downregulation [51]. For example, Rsp5-dependent degradation of the thiamine transporter Thi7, as well as its homologs, namely, the nicotinamide riboside transporter Nrt1 and the thiamine transporter Thi72, was observed. It was determined that Art2 is required for the proper degradation of all three transporters, whereas Art9 facilitates the proper ubiquitination of Nrt1 and Thi72 [51]. The latest study revealed a new role of α-arrestins in the control of phospholipid distribution and balance by the Art6- and Art3-dependent degradation of the Git1 transporter and the regulation of phosphatidyliniositol-3-phosphate levels [54]. Recently, data on yeast determined that isoflurane, an inhalation anesthetic used during surgical procedures [92], affects the dynamics of the Bap2 amino acid transporter’s ubiquitination and endocytosis in an Art2–Rsp5-dependent manner [52]. It is postulated that volatile anesthetics act on the function of synaptic membranes [93]. Yeast α-arrestins have also been shown to regulate GPCRs [48,49,74], possibly in both Rsp5-dependent and -independent mechanisms [48]. Overall, the latest studies suggest that transporter ubiquitination and endocytosis are preceded by molecular events affecting adaptors and transporters to acquire the ability to interact with each other.

### 3.2. Regulation of α-Arrestins

Several studies in yeast revealed that α-arrestins are regulated through post-translational modifications, especially phosphorylation and ubiquitination. Phosphoinhibition, the inhibition of protein activity by phosphorylation, seems to be a major mechanism responsible for the α-arrestin deactivation and blocking of α-arrestin-mediated protein trafficking. Multiple kinases including Npr1 (Nitrogen permeate reactivator 1) [45,86], a 5′-adenosine monophosphate-activated protein kinase (AMPK) homolog Snf1 (Sucrose non-fermenting 1) [67,94], Yck1/Yck2 (Yeast casein kinase homolog 1/2) [74], and Ypk1 (Yeast protein kinase 1) [95] were detected in yeast as directly related to α-arrestin phosphorylation and α-arrestin-dependent endocytosis. The α-arrestin phosphorylation occurs in response to nutrient availability or stress conditions, and their activity is coupled to signaling complexes such as the target of the rapamycin complex (TORC) 1/2 or AMPK (reviewed in [96]). In conditions such as nitrogen starvation or rapamycin treatment, TORC1 remains inactive, allowing for the TORC1 effector kinase Npr1’s activity and the α-arrestin’s phosphorylation [80,97]. In the case of nitrogen starvation, Nrp1 kinase phosphorylates several yeast α-arrestins, including Art1 [45], Art3 [53], and Bul1/2 [77,98], preventing ubiquitination and endocytosis of nutrient permeases, such as Gap1, Mup1, Can1, and Fur4 [45,77,97]. On the other hand, TORC1 activation (e.g., due to internal amino acid presence or cycloheximide treatment), results in hyperphosphorylation of Npr1 and thus α-arrestin activation followed by permease endocytosis [97,98].

The yeast Snf1 kinase is crucial for the metabolic adaptation of cells during glucose-limited conditions. Under glucose starvation, active Snf1 kinase phosphorylates and inactivates Art4 [94], resulting in the Hxt6 high-affinity glucose transporter’s stabilization [61,62]. Induced by 2-deoxyglucose, a toxic analog of glucose, downregulation of the Hxt1 and Hxt3 low-affinity glucose transporters also depends on Art4/Art7 in an Snf1-regulated manner [67]. When lactate is used as a carbon source, Snf1 impedes Art4 activity in the endocytosis of the lactate transporter Jen1, resulting in its stabilization at the cell’s surface and the stimulation of the lactate’s import [62,65]. Art1 has been found to undergo phosphorylation mediated by Pho85-dependent cyclins, Clg1, and Pho80 [26], and Art8 has been shown to be a target of protein kinase A (PKA) [63], whereas Art9 undergoes phosphorylation by casein kinase I (CKI) [74]. As most of the investigated α-arrestins were shown to bind 14-3-3 proteins [61,99], it seems to be a common mechanism of α-arrestin regulation, which results in the decrease in α-arrestin–Rsp5 complex formation and thus the inhibition of cargo endocytosis. The release of α-arrestins from their complexes with 14-3-3 proteins (and thus their activation) requires them to undergo dephosphorylation (reviewed in [100]). Several phosphatases have been found to interact with yeast α-arrestins, including Sit4 [51,78,97], calcineurin [49,54,56], protein phosphatase Z (Ppz) 1/2 [101], and protein phosphatase I (PPI) [62]. In general, dephosphorylation of arrestins causes their activation and function in the selective downregulation of permeases. Glucose-induced Art4 dephosphorylation by PPI is required for Jen1, Hxt1, and Hxt3 endocytosis [62,67]. However, Art4-dependent internalization of the Ste2 pheromone receptor requires dephosphorylation of the α-arrestin by the calcineurin phosphatase [49]. The dephosphorylation of Art6 by calcineurin mediates the endocytosis of the dicarboxylic amino acid transporter Dip5 [56] and the glycerophosphoinositol transporter Git1 [54]. It has been shown that Sit4 phosphatase has antagonistic effects on the Npr1 kinase in the regulation of the Art1- (and Bul1/2)-mediated endocytosis of amino acid transporters [45,79,97]. However, Art1 also undergoes dephosphorylation mediated by the Ppz1/2 phosphatase, promoting its activation in Mup1 and Can1 trafficking [101]. Although a regulation mechanism resembling the Snf1-dependent inactivation of Art4 has been observed for the human α-arrestin TXNIP [102,103] (revieved in [96]), which in the presence of glucose mediates GLUT1 and GLUT4 glucose transporter endocytosis [102,103], the phosphoregulation of human α-arrestins is highly undercharacterized, and it is tempting to speculate that additional similarities between the regulation pathways of yeast and human α-arrestins may exist.

The post-translational modification of an α-arrestin by ubiquitination seems to be important for its interaction with targets. Activation by the Rsp5-dependent ubiquitination of Art1, Art4, Art8, and Bul1 is relevant for these α-arrestins’ function in the endocytosis of various PM transporters [11,62,63,97]. It is not clear how ubiqitination regulates α-arrestins, and the functional significance of this post-translational modification remains to be elucidated. It was shown that the presence of ubiquitinated forms of Art1 (Art1-Ub) enhances downregulation of the Mup1 [104] and Can1 [11] transporters. However, Art1 ubiquitination seems to not be required for Art1-dependent Ste2 endocytosis [49]. Although Git1 endocytosis is Art6- and/or Art3-dependent, blocking of the Art6 or Art3 ubiquitin conjugation sites does not impair Git1 trafficking [54]. The ubiquitination of Art4 and Art7 is not absolutely required for Hxt1/3 low-affinity glucose transporter trafficking to the vacuole in the presence of 2-deoxyglucose [67]. Ubiquitinated forms of Bul1/2 act in Gap1 downregulation, when cells grow at a preferred nitrogen source, whereas ubiquitination is not important for the Bul1/2-dependent endocytosis of Gap1 in stress conditions [80,97]. Results of some studies suggest that the ubiquitination status of arrestins may depend on their (de)phosphorylation status, as phosphoinhibition and deubiquitination were observed at the same time. Lactate-medium-induced Art4 phosphorylation results in arrestin interaction with the 14-3-3 protein and a lack of Art4 ubiquitination [62]. The glucose-induced Art8 deubiquitination ties in with its phospho-regulated association with 14-3-3 proteins [63]. Several adaptors compete for Rsp5 in vivo. Ubiquitination increased the ability of the adaptors to compete effectively, which was diminished by a block of their ubiquitination. It was proposed that ubiquitination strengthens the association of α-arrestins to Rsp5 by binding to the ubiquitin-binding surface within the Rsp5 catalytic HECT domain, in addition to the interaction with the WW domain [104]. It has been established that the Ubp2 and Ubp15 deubiquitinating enzymes play an important role in α-arrestin regulation by limiting its ubiquitination, which influences the binding of adaptor to the Rsp5. This regulation provides the cycling of adaptors in response to distinct cellular requirements. Ubp2 and Ubp15 prevent hyperubiquitination and proteasomal degradation of α-arrestins, providing additional positive regulation of the adaptor–Rsp5 network [66,104].

Posttranslational modifications can also regulate α-arrestins at the level of subcellular localization or substrate interaction. Phosphorylated forms of Art1 reside in the cytosol and the Golgi apparatus, whereas dephosphorylation results in their association with PM [26]. Phosphomimetic mutation of Art1 impedes its interaction with Mup1 [101]. The non-ubiquitinable mutant of Art1 was stabilized in the cytosol [11]. Taken together, the reversible nature of (de)phosphorylation and (de)ubiquitination modification is an excellent regulatory mechanism for the directing of α-arrestin–Rsp5 complexes towards only a specific subset of cognate targets at the PM.

### 3.3. α-Arrestin–Substrate Interaction

A still poorly understood mechanism is how α-arrestins physically interact with their membrane substrates. Distantly related β-arrestins are known to bind their GPCRs and phosphoinositides through positively charged residues in both the N- and C-terminal arrestin domains [24]. For instance, the C-terminal arrestin domain of human β-arrestin 2 harbors a positive amino-acid-residue-enriched binding site (K233, R237, K251) for both inositol hexakisphosphate and phosphorylated GPCRs [105]. A recent study on the endocytic sorting signals of nutrient permeases allowed the identification of a similar basic region in the structure of the yeast α-arrestin Art1. Initial predictions suggested that Art1 harbors a single N-terminal arrestin-like domain with the PY motif located distantly in the C-terminal tail [11]. Guiney et al. demonstrated that the endocytosis-inhibiting effect of substitution in the N-terminal acidic region (acidic patch) of the methionine permease Mup1 can be suppressed by the corresponding mutations of the C-terminal basic residues of Art1 (R653 and R660) [46]. The observations that the mutation of these residues completely abolishes the function of Art1 indicate that it may interact with its membrane substrate through its C-terminal tail [26], and not the arrestin domain in a β-arrestin-resembling manner [24]. However, further analysis of the linkage between the structure and function of Art1 revealed that the arrestin in fact might form a full arrestin fold utilizing its whole primary amino acid sequence [26]. According to the revised model of Art1’s structure, the arrestin fold is constituted by well-conserved regions, which are divided by disordered loops and tails (Figure 1B). The positively charged substrate binding region is localized in the C-terminal domain of Art1, mimicking the substrate binding regions of β-arrestins [24,26]. Moreover, the loops and tails of Art1 are likely to participate in cargo recognition and substrate specificity. The deletion of “loop 3”, which is in close proximity to the substrate binding region of Art1, had no negative effect on Mup1 endocytosis but inhibited the degradation of the arginine permease Can1 [26]. The remaining loops and tails may be required for regulation of the general activity of arrestin, as their deletions combined severely hindered the degradation of both Mup1 and Can1, as well as decreased the ability of Art1 to bind to the PM [26]. The analysis of other yeast α-arrestins revealed similarities in fungal arrestins’ structural organization, as poorly conserved regions containing phosphorylation sites separating conserved regions were found. For instance, Art4 was predicted to possess two spacing loops, and their individual deletion distinctly affected Art4’s function, further reinforcing the hypothesis that the disordered loops may be involved in the substrate binding or specificity of α-arrestins [26]. The importance of the α-arrestin C-terminal basic motif in α-arrestin–Rsp5 binding to the “acidic patch” of the substrate and its further ubiquitination on a nearby lysine residue were determined for Art1 and Art2 in the case of Mup1 and Can1 endocytosis [46,47,106]. However, the C-terminal basic region of Art1 interacts with the N-terminal acidic patch of Mup1 (D43-L54), whereas the C-terminal basic motif of Art2 binds to the C-terminal acidic patch of Mup1 (D549-D555) [47]. Acidic amino acid sequences important for ubiquitination and/or endocytic degradation were also found in Lyp1, Tat1 [47], Jen1 [65], and Acr3 [57]. This suggests that the electrostatic interaction of the basic regions in the α-arrestin’s C-terminal half with acidic motifs in the substrate can serve as the recognition signal directing arrestin to the specific cargo.

Subcellular localization determines interactions with different pools of proteins. Similarly to their animal homologs, yeast α-arrestins have been shown to localize in various subcellular compartments (Table 1), indicating additional roles they may perform. Most of the α-arrestins localize to the cytosol, the PM and the TGN (trans-Golgi network). The relocalization of Art1 from the cytosol or the TGN to the PM under endocytic signals was observed [26,45]. Art8 has been shown to localize to the nucleus and act as a transcriptional repressor involved in carbon source metabolism regulation, e.g., it participates in the *GAL* and *CYC1* genes’ repression [71]. Both Bul1 and Bul2 proteins have been independently demonstrated to function in nucleus-specific processes related to the DNA damage response [107,108]. Art9, on the other hand, localizes primarily to the cytoplasm and, to some extent, the nucleus, when it is unable to bind the endosomal sorting complexes required for transport (ESCRT) machinery, although its nuclear role remains uncharacterized [73]. These observations imply that α-arrestins may perform other, poorly characterized functions in yeast cells aside from endocytosis regulation. Altogether, α-arrestins are conserved from yeast to humans, and many functional and structural similarities between fungal and animal arrestins have also been observed; thus, yeast *S. cerevisiae* serves as a great model for α-arrestin research.

## 4. Non-Arrestin Rsp5 Adaptors

Yeast α-arrestins are not the only PY-containing adaptors competing for Rsp5. Yeast α-arrestins play a key role in the PM proteostasis’s maintenance, as they cooperate with the Rsp5 ligase and compete with the other PY-motif-containing the non-arrestin adaptors Bsd2 [109], Tre1-2 [109], Rcr1-2 [110], Ear1 [111], Ssh4 [111], Hua1 [112], Sna3 [113], and Rup1 [114], constituting a complex adaptor–Rsp5 network (Figure 1D). Bsd2 is a membrane protein that contains the PY motif and mediates the Rsp5-dependent ubiquitination and vacuolar degradation of the manganese transporter Smf1-2 and the vacuolar proteins Cps1 and Phm5. Interestingly, Smf1 sorting requires first the sequential assembly of the Bsd2–Rsp5 complex and then the Tre1–Bsd2–Rsp5 complex; however, Tre1 is not involved in Phm5 degradation [109,115]. It was shown that the proper ubiquitination and sorting into endosomes/multivesicular bodies (MVB) of Phm5, as well as Gap1, the iron/siderophore transporter Sit1, and Fur4, requires the action of other Rsp5 adaptors, Ssh4 and Ear1, the lack of which causes the accumulation of those transporters in the vacuolar membrane. However, the vacuolar targeting of Smf1 does not depend on Ear1/Ssh4, again suggesting the specificity of the adaptor–Rsp5p complex toward the substrate [111,116]. Moreover, further studies indicated distinct localization and functional sites for Ear1–Rsp5 and Ssh4–Rsp5 at the endosome and vacuolar membrane, respectively [117]. The new data describe two paralogous transmembrane Rsp5 adaptors, Rcr1 and Rcr2, localized in the PM and vacuoles, respectively. It was shown that upon exogenous calcium treatment, Rcr1 ubiquitinates and downregulates the chitin synthase Chs3 [110]. Similarly, Sna3 was shown to cooperate with Rsp5 in the process of the ubiquitination and sorting of Mup1 in response to nutrient stress, displaying a partial redundancy with Art1 [113]. Rup1, on the other hand, mediates physical interactions between Rsp5 and the deubiquitinating enzyme Ubp2, which is crucial for the regulation of the Rsp5’s activity [114,118]. Interestingly, the Rup1–Rsp5–Ubp2 complexes were shown to localize to the MVB and interact with the Hse1 protein, a component of the ESCRT-0 complex [112,114]. This interaction was shown to require another PY-containing adaptor of Rsp5, Hua1, which was thus described as being involved in the regulation of the sorting efficiency into the MVB pathway [112].

## 5. Function of Human α-Arrestin Family

### 5.1. Unique Roles of Human α-Arrestins

Despite the general similarities in their structure and subcellular localization and a partial overlap in the functions performed by yeast and human α-arrestins, many aspects of their biology noticeably differ and still await elucidation. For instance, TXNIP is the only α-arrestin known to regulate the redox state in cells by binding and negatively regulating thioredoxin (TRX), as well as inducing apoptosis in the intrinsic mitochondrial pathway upon various stress stimuli [42,119]. Other studies hint towards the ability of these proteins to promote PM protein endocytosis in a NEDD4-independent manner, possibly through direct interaction with clathrin [103]. Even though several yeast α-arrestins have been also reported to promote endocytosis in *rsp5*-deficient cells, it seems that they target cargo proteins in the clathrin independent endocytosis (CIE) pathway rather than interact with clathrin in a human-α-arrestin-resembling manner [48]. 

The α-Arrestin ARRDC1 has been demonstrated to recruit the endosomal sorting complex required for transport of the (ESCRT)-0 complex subunit TSG101 to the PM and cooperate in the release of extracellular ARRDC1-mediated microvesicles (ARMMs) [41,120]. The interaction between ARRDC1 and TSG101 requires a conserved C-terminal PSAP motif of ARRDC1 to be bound by the ubiquitin E2 variant (UEV) domain of TSG101 [41], which highly resembles the mechanism by which the human immunodeficiency virus (HIV)-1 Gag protein recruits TSG101 to the PM, resulting in viral budding [121].

The studies on divalent metal ion transporter (DMT1) turnover implicated a novel mechanism of PM transporter abundance regulation by animal α-arrestins. Mackenzie et al. demonstrated that ARRDC1 and ARRDC4 regulate DMT1, although instead of canonically facilitating its lysosomal degradation, they mediate its release in extracellular vesicles (EVs) through distinct arrestin-specific mechanisms [31]. Both ARRDC1 and ARRDC4 were shown to promote DMT1 ubiquitination through NEDD4-2 ubiquitin ligase recruitment at the PM [31]. ARRDC1 was shown to promote DMT1 exocytosis through the mechanism mentioned above, whereas the ARRDC4-mediated sorting of DMT1 into EVs was speculated to rely on an ESCRT-independent viral budding pathway [31]. Altogether, ARRDC1 and ARRDC4 were proposed to utilize highly conserved membrane budding mechanisms in order to fulfill a novel role in the regulation of PM transporters, which was not shown so far in yeast α-arrestin homologs. Further investigation revealed that ARRDC4-mediated EVs are particularly produced in murine epididymal epithelial cells, and the global loss of *ARRDC4* expression in mice resulted in a decrease in male fertility due to changes in the sperm proteome and the loss in EV production [122]. 

### 5.2. α-Arrestins as Regulators of GPCRs

On the grounds that GPCRs’ regulation remains a hallmark role of β-arrestins, human α-arrestin involvement in that process has been investigated. Intriguingly, studies on GPCR β2-adrenergic receptor (β2-AR) turnover have contributed to the proposition of two competitive hypotheses for β2-AR regulation by α-arrestins. In 2001, Shenoy et al. showed that upon agonist stimulation, β-arrestin-2 undergoes dephosphorylation and binds activated β2-AR, which results in the arrestin’s ubiquitination and the subsequent endocytosis of the β2-AR–arrestin complex [123]. Additionally, β-arrestin-2 was initially proposed to recruit the NEDD4 ligase to the internalized receptor and thus facilitate its ubiquitination, allowing its further endosomal sorting and lysosomal degradation [124]. In 2010, however, Nabhan et al. identified the α-arrestin ARDDC3 as a crucial protein required for proper β2-AR degradation, although the presented results did not show a direct role of ARRDC3 in receptor internalization [125]. Since both β-arrestin-2 and ARRDC3 were shown to be required for proper β2-AR downregulation, it was proposed that these proteins may cooperate in β2-AR regulation [125]. Additional studies demonstrated that ARRDC3 interacts not only with β2-AR, but also the β3-adrenoreceptor (β3–AR), suggesting a general role of ARRDC3 in the regulation of β-adrenergic signaling [126]. Further investigation of the role of the α-arrestins ARRDC3 and ARRDC4, in β2-AR and vasopressin-2 (V2) receptor downregulation, respectively, suggested that α-arrestins might act coordinately with β-arrestins at the early steps of endocytosis to recruit the NEDD4 ligase to activated GPCRs and thus promote their ubiquitination, internalization, endosomal sorting, and lysosomal degradation [39]. Based on contradictory results, however, Han et al. proposed another hypothesis in which β-arrestin-2 remains the primary adaptor protein responsible for the initiation of clathrin-mediated β2-AR endocytosis, as well as the subsequent NEDD4 recruitment and receptor ubiquitination [7]. In this model, ARRDC3 (possibly together with ARRDC2 and ARRDC4) is a secondary adaptor localized at the endosomes, where it binds internalized β2-AR–NEDD4 complexes and mediated endosomal sorting of cargo [7].

Importantly, there is evidence for heterodimerization between both α- and β-arrestin families, as overexpression of ARRDC3 and ARRDC4 led to their co-immunoprecipitation with β-arrestins [39]. The study on the role of α-arrestins in the Notch receptor’s downregulation also suggested a cooperative activity of α- and β-arrestins [33]. ARRDC1 was demonstrated to negatively regulate the Notch signaling pathway together with β-arrestins by cooperative recruitment of the ITCH NEDD4-type ubiquitin ligase to the Notch receptor, resulting in its ubiquitination and lysosomal degradation [33]. Moreover, ARRDC1 was co-immunoprecipitated with β-arrestin, and overexpression of the PY-motif-lacking ARRDC1 mutant (which is unable to bind the ITCH ligase) resulted in a significant inhibition in the Notch receptor’s ubiquitination and degradation [33]. In view of this, β-arrestins are thought to heterodimerize through their arrestin domains with the α-arrestin ARRDC1, which can directly bind the ligase [33]. The study mentioned above not only indicates the role of α-arrestins in the degradation of the Notch receptor and thus in the regulation of developmental processes in animals, but also supports the idea of the cooperative functioning of both arrestin families in receptor downregulation. These results strongly support the hypothesis that α-arrestins are indeed involved in PM receptors’ regulation, including GPCRs (at least at the endosomal level). The involvement of human α-arrestins in endosomal sorting of other GPCRs has been observed as well. The internalization, sorting at the endosomes and lysosomal degradation of the protease-activated receptor-1 (PAR1) depends on the activity of the ALG-interacting protein X (ALIX), an endosomal adaptor protein, which is responsible for the ubiquitin-independent linking of activated PAR1 to the ESCRT-III complex [127]. However, it was demonstrated that the proper functioning of ALIX in this process requires the WW2 NEDD4-type ubiquitin’s ligase-dependent ubiquitination in an active PAR1-dependent manner, and this process involves α-arrestins [127]. In view of ARRDC3 co-localizing with PAR1 and ALIX on endosomes, as well as the fact that the diminution of ARRDC3 precludes PAR1–ALIX interaction and PAR1 lysosomal targeting, ARRDC3 was proposed to regulate PAR1 degradation through control of ALIX’s ubiquitination by the WW2 ligase [127]. 

Given the important role of GPCRs in a plethora of cellular processes, β-arrestins, which are known regulators of GPCR activity, have been repeatedly proposed as potential therapy targets in many diseases. For instance, Komatsu et al. have recently discussed an approach for treatment of psychiatric disorders based on developing selective drugs—biased GPCRs ligands—which are able to activate GPCRs, although they modulate only selected downstream signaling pathways (including β-arrestin-dependent pathways), allowing the limitation of potential side effects and thus constituting a promising tool for therapies against mental disorders [128]. Such strategies have already been proposed for the regulation of dopamine transmission and signaling, whose dysfunction is crucial for the development of dyskinesias as in Parkinson’s disease (PD), and the progression of other central nervous system (CNS) disorders, such as Alzheimer’s disease (AD) and schizophrenia [129]. Considering the many functional similarities and possible cooperation between α- and β-arrestins, the former emerges as another promising target in therapy based on the activity of GPCRs. Nevertheless, devising such therapies would require a greater understanding of their involvement in the regulation of GPCRs; thus, further research is needed.

### 5.3. α-Arrestins in Cancer Research

The α-arrestin TXNIP, thanks to its pro-apoptotic properties, is an important tumor suppressor (reviewed in [20]). Indeed, several studies demonstrated that the level of TXNIP is significantly decreased in human cancers in general [20,130,131]. It has been demonstrated that TXNIP is downregulated in tumors due to both epigenetic silencing [132] and proteolysis, as the NEDD4-type ubiquitin ligase ITCH interacts with TXNIP through its C-terminal PY motifs and targets it for proteasomal degradation [34]. Recent studies have revealed that other human α-arrestins are also involved in the regulation of carcinogenesis. The yeast two-hybrid analysis demonstrated that the least characterized human α-arrestin, ARRDC5, is one of the 226 potential interactors of Hsp27, a stress-induced chaperone, which is often upregulated in tumors. The study predicted ARRDC5 to be a part of an interaction network involved in the “regulation of ubiquitin ligase activity during mitotic cell cycle” [133]. On the other hand, ARRDC2 has been found to be strongly upregulated in brain ependymoma, indicating its oncogenic potential [8]. However, overexpression of the *ARRDC2* gene alone did not drive neoplastic transformation in murine cerebral neural stem cells, suggesting that the oncogenic potential of ARRDC2 may depend on a broader genetic and/or paracrine context [8]. Another human α-arrestin, ARRDC3, has been shown to inhibit cell proliferation when overproduced [19]. ARRDC3 has been suspected to act as a potential metastasis suppressor in many human cancers [134] and as a diagnostic and prognostic marker for ovarian cancer [38]. On the grounds that integrin secretion in EVs seems to be pivotal for pre-metastatic niche formation in organ-specific cells [135], and ARRDC3 has been found to negatively regulate integrin β4 (ITG β4) [134], the α-arrestin has been hypothesized to negatively regulate metastases. Indeed, it has been recently confirmed that ARRDC3 is pivotal for metastasis suppression in triple negative breast cancer (TNBC) cells, where ITG β4 undergoes endosomal recycling and exocytosis, allowing cancer cell invasion [32]. ARRDC3 has been demonstrated to prevent ITG β4 recycling from endosomes [32,134]. These observations indeed render ARRDC3 as a potential therapy target in metastatic breast cancer. In fact, it has been already shown that KPT-185 and selinexor, two selective inhibitors of nuclear export (SINE) targeting exportin-1, restrain TNBC’s growth and invasiveness by restoring the epigenetically silenced expression of *ARRDC3* at both the mRNA and protein level [136]. The role of ARRDC3 in tumor suppression, however, seems not to be limited to the regulation of ITG β4 turnover. As mentioned earlier, ARRDC3 is known to participate in the negative regulation of the PAR1 receptor [127], which is overproduced in breast cancer cells [137], and aberrations in its signaling, for instance, due to dysregulation of lysosomal trafficking, influence metastasis [138]. Indeed, restoration of ARRDC3 synthesis in breast cancer cells improved ligand-induced degradation of PAR1, inhibited its persistent activation and thus attenuated PAR1-dependent cancer cell invasiveness [139]. Moreover, augmented *ARRDC3* expression inhibited GPCR-dependent activation of the Hippo signaling pathway, which is considered crucial for cancer progression [140]. Two major effectors of the Hippo pathway are the Yes-associated protein (YAP) and a transcriptional co-activator with PDZ-binding motif (TAZ) oncogenes, whose prolonged activity results in aberrations in cell proliferation, the suppression of apoptosis, and general cancer initiation and progression [141,142]. Both YAP and TAZ possess WW domains [142]. ARRDC3 was demonstrated to inhibit PAR1-dependent Hippo signaling in breast cancer cells in a novel, PAR1-degradation-independent manner, by directly interacting with TAZ and inhibiting its activity [143]. Similarly, α-arrestins have been demonstrated to bind and promote degradation of YAP in colorectal cancer cells, consequently increasing their susceptibility to the chemotherapeutics doxorubicin, 5-fuorouracil, and cisplatin [144]. Intriguingly, in clear cell renal cell carcinoma, ARRDC3 and ARRDC1 have been shown to be involved in YAP destabilization [145]. Both α-arrestins bind YAP through WW–PY motif interaction and recruit the ITCH ligase for the ubiquitination and subsequent degradation of YAP [145]. Considering these observations and the latest advances in cancer research, which indicate that all cancer types could be divided into binary YAP^on^ and YAP^off^ classes based on YAP expression or silencing, respectively [146], the α-arrestin-dependent regulation of YAP seems to be a promising target of future therapies for multiple human malignancies.

α-Arrestins may also serve as promising targets in cancer treatment on the grounds that regulation of their activity may negatively affect the global metabolism of tumor cells. For instance, given that the (i) GLUT1 transporter has been shown to be highly upregulated in cancer cells, as it is considered crucial for the maintenance of high glycolysis rates in tumors [147]; (ii) the inhibition of GLUT transporters has been shown to aggravate cancer cell growth [148]; (iii) high levels of TXNIP negatively regulate GLUT1 at both the protein and transcriptional level [102]; and (iv) 2-deoxyglucose has been shown to promote degradation of the GLUT homologs Hxt1 and Hxt3 in yeast through its impact on α-arrestins [67], TXNIP might serve as a potential therapy target, which could negatively affect global cancer cell metabolism when upregulated. Additionally, in the face of the growing problems of cancer drug resistance, which depend on an increased drug efflux or decreased drug uptake [149], a therapy approach based on PM transporter regulation by α-arrestin targeting could be potentially employed. However, little is still known about the α-arrestin-dependent regulation of PM proteome in human cells, therefore potential therapies require more knowledge of novel α-arrestin substrates.

Another postulated α-arrestin-based approach for cancer treatment utilizes the idea of employing EVs as endogenous drug carriers. The ability of ARRDC1 to selectively load certain classes of macromolecules into ARRDC1-mediated microvesicles (ARMMs) has been recently tested. It was demonstrated that the p53 protein fused to the C-terminus of ARRDC1 was effectively packaged into ARMMs, and the fusion protein consequentially increased the expression of p53 target genes in recipient cells lacking intrinsic p53 and promoted apoptosis in irradiated p53-null mice otherwise resistant to apoptosis [150]. It would be interesting to see whether another human α-arrestin, ARRDC4, which has been recently reported to facilitate EVs formation [31], is capable of selectively loading EVs with therapeutic macromolecules similarly to ARRDC1. 

### 5.4. The Role of α-Arrestins in Cellular and Tissue Metabolism

On the grounds that yeast α-arrestins are key players in the abundance regulation of various PM transporters, including carbon source compounds [13,65,67], amino acids [13,46,53], metals [13], metalloids [57] or vitamin transporters [51], α-arrestins indirectly regulate the global cell metabolism [100]. Considering the structural and functional similarities between yeast and human α-arrestins, as well as the recently discovered unique properties of the latter, one may suspect that human α-arrestin family members would fulfill at least a similar role. Indeed, a growing amount of evidence implies that mammalian α-arrestins are involved in the regulation of metabolisms on both the cellular and organismal levels. 

Studies in several mammalian models implied a role for α-arrestins in muscle development and metabolism. For instance, ARRDC2 has been observed to be significantly upregulated in the semitendinosus muscle tissue of lambs born to overfed ewes [151]. On the other hand, it has been shown that in catabolic-state-induced skeletal muscle (SM) atrophy, the knockout (KO) of the muscle-specific ring finger 1 (MuRF1) ubiquitin ligase results in the abolition of *ARRDC2*’s gene expression, indicating that *ARRDC2* might be a novel gene involved in glucocorticoid response [152]. Additional studies in mice revealed that the expression of *ARRDC2* in SM significantly increases in response to exercise. In fact, *ARRDC2/3* are consistently down- or upregulated in response to anabolic (nutrient uptake, mechanical overload) and catabolic (decreased testosterone levels due to castration, aerobic exercise) stimuli [153]. Altogether, mammalian α-arrestins, particularly ARRDC2/3, are suitable candidates for regulators of muscle metabolism, possibly due to their involvement in the regulation of several signaling pathways, including β-adrenergic and corticosteroid signaling pathways. Several α-arrestins have already been identified as pivotal regulators of glucose metabolisms as well. The most prominent example is TXNIP, the expression of which is induced in fasting mice [126] and is also downregulated in response to insulin and upregulated by glucose [154]. The α-arrestin has been previously associated with the inhibition of glucose importing and the promotion of lactate extrusion from human cells [40]. Recent studies showed that TXNIP, similarly to its yeast homologs, is localized to the PM, where it mediates the endocytic downregulation of GLUT1 and GLUT4, and thus regulates glucose uptake in fat and muscle tissues [102,103]. Although the α-arrestin seems to promote endocytosis of GLUT1 and GLUT4 through interactions with clathrin [102], the mechanism controlling the activity of TXNIP in this process shares certain characteristics with the mechanism regulating the α-arrestin Art4 involved in sugar homeostasis maintenance in yeast cells (reviewed in [96]). Phosphorylation of TXNIP was shown to abolish its interaction with phosphoinositide, causing dissociation of TXNIP from the PM and preventing the GLUT–TXNIP complex’s formation, which leads to a significant decrease in GLUT endocytosis and an accelerated glucose influx [103]. The observations clearly demonstrate that TXNIP is a key regulator of glucose uptake with GLUT transporter downregulation in both a glucose-dependent negative feedback loop and an insulin signaling-dependent manner. These observations make TXNIP a promising therapeutic target for obesity and diabetes type I and type II treatment [15], especially since it has been previously shown that mice devoid of TXNIP, despite having an increased fat mass, are indeed protected from developing insulin resistance [155], and that TXNIP depletion protects mice against β cell apoptosis [119]. Aside from the glucose metabolism, TXNIP has been identified as a potent lipid metabolism regulator (reviewed in [156]). In human cells, their glucose metabolism is controlled not only by TXNIP, but also by ARRDC4—overexpression of both α-arrestins provided decreased glucose uptake and increased lactate efflux in human skin fibroblasts [40]. In several human tissues, including SM and pancreatic β-cells, the glucose-induced expression of *ARRDC4* and *TXNIP* is controlled by the MondoA transcription factor [157,158], whose target genes provide suppression of glucose uptake [159]; therefore, they act as insulin signaling pathway suppressors. ARRDC3 has been recently discovered to control glucose and insulin signaling and to be an important regulator of the liver metabolism, as it functions in a negative feedback loop regulating the insulin receptor and thus the insulin response in mice. ARRDC3–IR interaction requires ARRDC3 to be phosphorylated in an insulin-dependent manner [16]. In concordance with previously reported observations that (i) a rare haplotype in the *ARRDC3* locus is linked to male obesity in humans, (ii) global KO of *ARRDC3* in mice protects them against age-induced obesity, insulin resistance, and hepatic steatosis, and (iii) ARRDC3 seems to be involved in modulation of adipose tissue functioning through the regulation of β-adrenergic signaling [126], the presented findings suggest that ARRDC3 may be a promising target for obesity and/or diabetes treatment.

### 5.5. α-Arrestins as Immune Response Regulators

For many years, the involvement of α-arrestins in the mechanisms underlying immune responses has been rather vague. Recent studies, however, provide a growing amount of evidence for α-arrestins acting as key players in providing protection against pathogens. For instance, Rauch and Martin-Serrano established that several α-arrestins might be recruited to sites of viral buddying in HeLa cells [160]. ARRDC1, together with a cytoplasmic variant of the WWP1 NEDD4-type ubiquitin ligase, were demonstrated to relocate from punctate structures to the PM when co-expressed with the Ebola virus matrix protein VP40 [160].

Viral infection may affect the expression of α-arrestins. *ARRDC2* has been identified as one of the genes upregulated in human lung epithelial cells upon infection with respiratory syncytial virus [161], and a recent study based on machine learning proposed the *ARRDC2* gene as a potential target of the miRNA of the human endogenous retrovirus K-113 [162], which is often upregulated in breast cancer [163]. *ARRDC3* KO in HeLa cells resulted in a significant decrease in their vulnerability to HPV16 pseudovirion infection, implying a crucial role of α-arrestin during early steps of HPV infection [164].

α-Arrestins have been long proposed to participate in innate immune responses. TXNIP, for one, has been associated with the nucleotide binding and oligomerization domain (NOD)-like receptor protein 3’s (NLRP3) inflammasome activation by means of the NF-κB signaling pathway’s regulation, e.g., in response to oxidative stress [165] and uric acid stimulation [43]. Several studies, however, presented results contradictory to the postulates mentioned above [166,167]; thus, the role of TXNIP in inflammasome activation remains pending. A novel role for ARRDC4 in the innate immune response to viral infections has been recently proposed. Meng et al. demonstrated that the expression level of ARRDC4 was visibly increased in patients suffering from hand, foot, and mouth disease (HFMD), which is mainly caused by enterovirus 71 (E71) infection [168]. Further investigation demonstrated that the ARRDC4-mediated ubiquitination of the MDA5 receptor seems to be an important regulation mechanism required for the oligomerization and activation of this receptor upon viral RNA binding, which consequently allows the downstream innate immune signaling pathway’s activation and proper response to E71 infection [168]. Additionally, a recent study suggests that ARRDC1 may be (at least indirectly) involved in the activation of T lymphocytes by recruiting endocytic machinery for membrane budding; thus, α-arrestins might be involved in the adaptive immune response as well [169].

### 5.6. α-Arrestins in Brain and Neurodegenerative Diseases

Considering their documented expression in brain tissue [35,170,171], as well as the multitude of their cellular functions mentioned above, the arrestin clan members have been frequently associated with central nervous system (CNS) disorders. Indeed, growing evidence indicates that both β- and α-arrestins might play a crucial role in the pathogenesis of several neurodegenerative diseases (NDs), psychiatric disorders (PDs), and substance use disorders (SUDs). Due to the notably high cerebral expression of GPCRs (such as dopamine and serotonin receptors) [172], as well as the immense importance of GPCRs’ signaling for proper brain functioning, the ability of arrestins to regulate GPCRs have seen them be proposed as targets in therapy against major brain pathologies. As the functions of β- and α-arrestins seem to at least partially overlap, it is tempting to speculate that α-arrestins may play important roles in CNS disorders as well. Various neurodegenerative diseases, especially AD, develop in conditions of age-linked chronic neuroinflammation and oxidative stress [173]. As was mentioned earlier, a growing number of evidence links the α-arrestin TXNIP to NLRP3-dependent inflammation [43,165,174]. Recently, it was demonstrated that TXNIP directly promotes NLRP3 inflammasome hyperactivity in aging mice [174]. TXNIP upregulation was correlated with the decrease in the anti-aging protein klotho, as well as an increase in NLRP3 inflammasome components. Either KO of TXNIP or treatment with TXNIP-repressing pharmaceutical verapamil resulted in the repression of NLRP3 inflammasome assembly in elderly mice [174]. Additionally, the increase in TXNIP levels corresponded to the downregulation of thioredoxins [174], whose involvement in redox homeostasis regulation renders them potent neuroprotective proteins [175]. Given the involvement of TXNIP in both neuroinflammation and oxidative stress, α-arrestin might serve as a substantial target for AD treatment. Indeed, it has been demonstrated that substances such as verapamil, Salidroside, estrogen, and D1-3-n-butylphthalide (D1-NBP) can negatively regulate TXNIP and/or its interaction with NLRP3 inflammasome [175]. Moreover, an increasing amount of research suggests that many natural phytochemicals, such as flavonoids and phenols, act not only as antioxidant and anti-inflammatory agents, but also directly interact with TXNIP, reduce its production and inhibit its interaction with NLRP3 inflammasome components [176]. It was found that the level of TXNIP was elevated in the hippocampus of AD mice, which was accompanied with TRX inhibition. In vitro treatment of SH-SY5Y neuroblastoma cells with Aβ(1-42) also resulted in TXNIP overproduction and TRX inhibition [177,178]. It suggests that TXNIP inhibition may have a neuroprotective effect. Besides AD, the proapoptotic properties of TXNIP have been found to be crucial for neuronal apoptosis in several other brain pathologies, including prediabetic neuropathy, subarachnoid hemorrhage and diabetes-linked PD [171,179,180,181,182]. Overproduction of TXNIP in transgenic mice or transfected HEK cells induced α-synuclein accumulation [183]. The role of TXNIP in brain and neurodegenerative diseases has been profoundly reviewed in [18].

The role of α-arrestins other than TXNIP in brain pathologies remains poorly documented, and only few connections with CNS disorders have been made so far. As was mentioned before, α-arrestins are involved in the regulation of the degradation of Notch receptors [33], and therefore they participate in the modulation of the Notch signaling pathway, which is crucial for both neural development and adult brain functioning [184]. A novel α-arrestin-dependent mechanism of Notch pathway regulation has been recently identified. It was observed that multiple Notch pathway components specifically localize to ARMMs together with ARRDC1 and the ITCH ubiquitin ligase [185]. Disruption of the *ARRDC1* gene strongly decreased NOTCH2 secretion, indicating that this process is ARRDC1-dependent [185]. Moreover, the EVs containing secreted NOTCH2 could be successfully transferred to recipient cells, where γ-secretase provided for its further proteolytic processing, resulting in the activation of the Notch target genes [185]. These observations strongly suggest that the α-arrestin ARRDC1 provides a novel mechanism allowing for the modulation of noncanonical, long-distance Notch signaling in neuronal tissue.

An increasing number of studies suggest that α-arrestins may be also involved in the molecular response to psychoactive substances and hormones in brain tissues. A study on gene expression changes in the prefrontal cortex of mice treated with hallucinogen lysergic acid diethylamide (LSD) recognized *ARRDC2* as an LSD-responsive gene, which is partially affected by 5-HT_2A_ serotonin receptor activity, indicating the potential role of ARRDC2 in molecular mechanisms responsible for LSD-induced changes in animal behavior [186]. Similarly, *ARRDC2* mRNA has been observed to be strongly upregulated in the hippocampus of rats upon stimulation with a single high dose of psilocybin, which is a 5-HT receptor agonist and a potential drug in the treatment of psychiatric disorders [187]. This result renders *ARRDC2* as an important psychedelic-drug-responsive gene, and given the involvement of these substances in neural plasticity [188], α-arrestin may be involved in this process as well. Moreover, similarly to the case of muscle tissue, studies on glucocorticoid signaling in the brain revealed that *ARRDC2* is a key target gene of glucocorticoids in both the hippocampus and hypothalamus in rodents [189] and pigs [190], although its role in corticosteroid responses remains elusive. Furthermore, in compliance with the observation that glucocorticoid concentrations may increase with age [191], studies on gene expression in cerebral tissues of aging mice revealed an upregulation of *ARRDC2* [192], although its role in the brains of aging mice remains uncharacterized.

A study on neural stem cell (NSCs) differentiation in a murine model demonstrated that the α-arrestin ARRDC3, which has been previously linked to cognitive deficiencies in humans [193], is a component of mouse NSCs’ regulatory networks [194]. In view of these observations, further inquiry of the role of ARRDC3 in the mechanisms regulating NSCs may lead to the amelioration of the previously proposed stem cell therapy against neurological disorders [195]. Furthermore, another study on miRNA–mRNA interactions demonstrated that *ARRDC3* mRNA is targeted by several miRNAs in a murine AD model [196]. Interestingly, the level of *ARRDC3* mRNA in mice was found to be noticeably increased in an age-dependent manner [196]. The beneficial, neuroprotective effect of flavonoids and phenols on the cognitive function of AD was proposed concerning the observation that flavonoids and phenols significantly inhibit TXNIP production (reviewed in [176]). It is noteworthy that many studies indicate the important role of the NEDD4-type ubiquitin ligases in neurodevelopmental disorders and neurodegenerative diseases (profoundly reviewed in [197]). It tempts speculation that α-arrestins as adaptors linking the ligase to substrates may participate in neurodegeneration associated with NEDD4-type ubiquitin ligases’ dysfunction. 

Collectively, the accumulated data suggest that α-arrestins are closely associated with CNS disorders, although further research is certainly required. In view of the functional similarities between α- and β-arrestins, which include GPCR regulation and PM protein downregulation, as well as the documented cooperation between the two protein families, it is highly probable that α-arrestins may participate in at least part of the cellular events accounting for the pathogenesis of brain diseases, and thus similar treatment strategies could be employed in the future.

## 6. Summary

α-Arrestins constitute a large family of proteins conserved from yeast to humans. Many functional and structural similarities between fungal and animal α-arrestins have been observed; thus, yeast *S. cerevisiae* serves as a great model for α-arrestin research. The predominant function of α-arrestins seems to be cooperation with the Rsp5/NEDD4 family of ubiquitin ligases in the regulation of endocytosis and the further sorting of various PM proteins, which links α-arrestins to the regulation of PM proteostasis, as well as various signaling pathways and global cellular metabolisms. However, α-arrestins have been shown to localize in various subcellular compartments, indicating additional functions they can perform. The molecular mechanisms of the selective substrate’s targeting and regulation of α-arrestins in response to different stimuli remain incompletely understood. Recent advances in fungal α-arrestin research may be crucial for the characteristics of their human counterparts. Despite poor characterization, mammalian α-arrestins have been linked to several health disorders such as diabetes, tumor progression, and neurodegenerative disorders, making them promising targets for future therapies. However, many aspects of α-arrestin’s biology remain vague, especially the mechanisms of selective substrate targeting and the regulation of activity; thus, α-arrestin research remains a challenge for future studies.

## Figures and Tables

**Table 1:** Yeast α-Arrestins.

Name	Systematic Name	Subcellular Localization	Posttranslational Regulation (Experimentally Determined)	Substrates	Identified Substrate Binding Sites
Art1/ Ldb19	YOR322C	Cytoplasm, PM, trans-Golgi network (TGN) [11,44], early endosomes [45]	Inactivated through Npr1-dependent [45] and Clg1-dependent [26] phosphorylation; activated through Pho80-dependent phosphorylation [26] and Rsp5-dependent ubiquitination [11]	Mup1 [11,26,46,47,48] Can1 [11,26,45,46], Tat2 [13], Fur4 [13], Lyp1 [11,26], Ste2 [48,49], Ste3 [48]	Basic residues in the C-terminal half of the protein (R653, R660) [46]
Art2/ Ecm21	YBL101C	Cytoplasm [44]	N/D	Mup1 [47], Can1 [47], Lyp1 [47], Ina1 [47], Smf1 [50] Tat2 [13,47], Fur4 [13], Thi7 [51], Thi72 [51], Nrt1 [51], Bap2 [52]	Basic residues in the C-terminal half of the protein (K664D, R665D, R666D, K667D) [47]
Art3/ Aly2	YJL084C	Cytoplasm [44], endosomes, TGN [53]	Monoubiquitinated at K392 (unknown function) [54]	Gap1 [53], Dip5 [55,56], Ste3 [48], Acr3 [57], Git1 [54], Ena1 [58], Put4 [59]	N/D
Art4/ Rod1	YOR018W	PM [60], GA, vacuole [61]	In the absence of glucose negatively regulated by Snf1-dependent phosphorylation; activated through PPI-dependent dephosphorylation and Rsp5-dependent activating ubiquitination [62]	Jen1 [62,63,64,65], Hxt6 [13,26,61,66], Hxt1 [61,67] Hxt3 [47,67] Acr3 [57], Ste2 [48,49], Stl1 [68], Gal2 [69]	The unspecified region at the N-terminal portion (amino acids 1-395) of the protein [61]
Art5	YGR068C	Cytoplasm [70]	N/D	Itr1 [13]	N/D
Art6/ Aly1	YKR021W	Endosomes, TGN [53]	Positively regulated by calcineurin-dependent dephosphorylation (56); monoubiquitinated at K391 (unknown function) [54]	Gap1 [53], Dip5 [55,56], Ste3 [48], Git1 [54]	N/D
Art7/ Rog3	YFR022W	Cytoplasm [70]	N/D	Hxt3 [67], Ste2 [48,49], Hxt6 [66]	N/D
Art8/ Crs2	YPR030W	Cytoplasm, nucleus [71]	Activated through Rsp5-dependent ubiquitination; inactivated through deubiquitination and PKA-dependent phosphorylation [63]	Smf1 [50], Fur4 [13], Tat2 [13], Hxt2 [63], Hxt3 [67,72], Hxt4 [63], Hxt6 [63], Hxt7 [63]	N/D
Art9/ Rim8	YGL045W	PM, cytoplasm, nucleus [73]	Requires Rsp5-dependent monoubiquitination for ESCRT recruitment [73]; CK1-dependent phosphorylation prevents the PM association [74]	Rim21 [73,74] Nrt1 [51], Thi72 [51], Pma1 [75], Ena1 [76]	N/D
Art10	YLR392C	Cytoplasm [44]	N/D	N/D	N/D
Bul1	YMR275C	Cytoplasm [44]	Nitrogen starvation causes inhibition through Npr1-dependent phosphorylation; activated trough Sit4-dependent dephosphorylation and Rsp5-dependent ubiquitination [77]	Jen1 [78,79], Gap1 [53,80], Ptr2 [81,82], Tat1 [83], Tat2 [13], Ctr1 [84], Put4 [82], Dal5 [82], Agp1 [85], Fur4 [13], Can1 [86], Gal2 [69]	N/D
Bul2	YML111W	Cytoplasm [44]	Nitrogen starvation causes inhibition through Npr1-dependent phosphorylation; activated trough Sit4-dependent dephosphorylation and Rsp5-dependent ubiquitination [77]	Fur4 [13], Jen1 [79], Gap1 [53,80], Ptr2 [81,82], Tat1 [83], Tat2 [83], Ctr1 [84], Put4 [82], Dal5 [82], Can1 [86]	N/D
Bul3	YNR069C/YNR068C	N/D	N/D	N/D	N/D
Spo23	YBR250W	N/D	N/D	N/D	N/D
